# Outcomes of AfroPHC, a WONCA Africa project

**DOI:** 10.4102/phcfm.v13i1.3116

**Published:** 2021-09-02

**Authors:** Shabir Moosa

**Affiliations:** 1 Department of Family Medicine and Primary Care, Faculty of Health Sciences, University of the Witwatersrand, Johannesburg, South Africa; 2 WONCA Africa, South Africa

The African Forum for Primary Health Care (AfroPHC) (https://afrophc.org/about/) was initiated by the African Region of Wonca, the World Organisation of Family Doctors in January 2020 (https://www.woncaafrica.org/), to bring together the leadership of all healthcare workers at the coalface in African primary care and ensure that we all have a voice in policy on primary health care (PHC) and universal health coverage (UHC) in Africa.^1^ We wanted to build an accountable team-based PHC for well-defined populations at scale in Africa.

The AfroPHC Statement was a milestone in setting out a vision for the group in its first workshop conducted during 9–11 September 2020. The African Forum for Primary Care (AfroPHC) consists of multidisciplinary PHC stakeholders from across Africa, who share a vision for African PHC service deliver: primary health care should be ‘comprehensive, accessible, high quality, responsive to local needs, in partnership with communities and delivered by strong teamwork, training and supportive supervision’. The Statement elaborates on the key principles of PHC as people-centred, with adequate human resources, capacity development, teamwork, inclusive leadership and advocacy.

A series of workshops have developed a remarkable consensus on building the PHC team in Africa. The AfroPHC language of multidisciplinary teams and the inclusion of all relevant healthcare workers, with everyone focused as a team on holistic patient-centred care, rather than fragmented task-shifting, were supported. A number of human resource challenges for PHC were raised, including strengthening resources and data for human resources (including varying nomenclature), the importance of training to manage patients’ psychosocial issues, the need to link individual care with multisectoral and population care, the importance of decentralised leadership focused on defined populations, and the need to include the private sector in human resources and UHC planning.

There is consensus on the need for comprehensive planning, supported by political commitment, good governance, an enabling environment and relevant skills mix. A useful framework presented at a workshop on human resource management for PHC in Africa is the working lifespan framework from World Health Organization (WHO)^2^: Entry (Preparing the workforce with planning, education and recruitment), Workforce (Enhancing worker performance with supervision, compensation, systems support and lifelong learning) and Exit (Managing attrition with migration, career choice, health and safety and retirement). All this is expected to lead to Workforce Performance (availability, competence, responsiveness and productivity).

The AfroPHC is not only looking to influence policy, but also to support collaboration between the various cadres of the PHC team in Africa. A workshop on the development of PHC clinicians in Africa shared lessons from training of family doctors, advanced nurse practitioners and clinical officers. Some useful lessons were the alignment of training (programmatic learning outcomes informing curriculum content, assessment and educational approach) with resources, and roles and responsibilities in the health system. The adult learning approach was central to decentralised training centres, workplace-based learning and online support. The training of clinical trainers and a national licensing examination were important. All advocated for educational frameworks and standards, recognition, collaboration and promotion of PHC in Africa. High level support from WHO and teamwork with WONCA Africa was valued in the educational space. Another workshop on supporting community health workers (CHW) as part of the PHC team in Africa reflected AfroPHC values. The highlights of WHOs Handbook on Social Participation for UHC were shared.^3^ Several CHW activists shared the useful work being performed by CHWs through linkages to communities. They also shared the challenges of CHWs as volunteers without recognition or payment, verticalised work, poor formal support, and poor linkages to facilities.

AfroPHC started off in January 2020 trying to build teamwork and trust across members of the PHC team in Africa. That trust is now formalised with AfroPHC launched in a General Meeting on 20 April 2021. An Executive Board was elected, with strong competence and diversity across gender, cadre, geography and language in Africa. This cohesion is paying dividends with AfroPHC (and WONCA Africa) being drawn into several forums as an organisation that stakeholders are eager to listen to, including WHO.

The next few workshops are critical to the theme of building PHC teamwork for Africa. We are planning a workshop on PHC in health professions education, jointly with the African Interprofessional Collaborative Practice and Education Nework (AfrIPEN). We need to ensure that teamwork happens without hierarchical competition in African PHC. We are also planning a workshop on PHC funding and payment systems in Africa, jointly with Strategic Purchasing – Africa Resource Centre (SPARC) and its African network of government officials in financing issues. The objective is to understand the dynamics of funding and the possible new payment systems being envisaged for PHC across Africa. Other policy issues that will be addressed in workshops till the end of 2021 are complexity, quality and safety, rural challenges, and digital health in African PHC.

AfroPHC will continue these workshops (building advocacy and networking) and collaboratively develop training, research, partnerships and chapters in regions and countries across Africa. Our vision is to see AfroPHC, and the ideal of people-centred PHC teamwork, being evident on the ground in clinics and communities across Africa. WONCA is playing a key part in AfroPHC and the outcomes are impressive.
